# Successful transcatheter aortic valve implantation for severe aortic stenosis of a bicuspid valve with situs inversus totalis guided by advanced image processing: a case report

**DOI:** 10.1093/ehjcr/yty049

**Published:** 2018-05-04

**Authors:** Pavel Overtchouk, Cédric Delhaye, Arnaud Sudre, Thomas Modine

**Affiliations:** Department of Cardiology and Cardiovascular Surgery, Centre Hospitalier Regional et Universitaire de Lille (CHRU de Lille), 2 Avenue Oscar Lambret, Lille, France

**Keywords:** TAVI, TAVR, Image processing, Situs inversus, Bicuspid, Case report

## Abstract

**Introduction:**

Transcatheter aortic valve implantation (TAVI) can be challenging in case of complex anatomy such as bicuspid aortic valve stenosis or situs inversus. This report illustrates a successful procedure in a patient with both conditions after careful pre-operative planning and per-operative guidance by means of a novel software.

**Case Presentation:**

We report the case of a 71-year-old man that presented a type 0 bicuspid aortic valve stenosis and situs inversus. We performed transfemoral TAVI using the Edwards Sapien 3 transcatheter heart valve (THV) and a novel software that provides insight for patient anatomy through computed tomography (CT) extracted three-dimensional reconstruction before the procedure aiming at improving THV positioning during the procedure through fusion of a CT-extracted virtual aortic annulus on the fluoroscopy screen and enhancing of calcifications that can be considered as anatomical landmarks. The Edwards Sapien 3 THV was accurately implanted in a supra-annular fashion. Post-operative echocardiography showed an excellent result without any para-prosthetic leak, and the patient reported symptom improvement.

**Discussion:**

During TAVI the angiographic appearance of the cusps of a bicuspid aortic valve is irregular and asymmetric, which can lead to malpositioning, paravalvular regurgitation, and increased rates of pacemaker implantation after TAVI. Furthermore, usual anatomical landmarks can be even more disturbed by the situs inversus totalis. We believe that pre-operative three-dimensional reconstruction and per-operative fluoroscopy image processing, provided by software such as the one presented in this case report, can provide precious guidance for TAVI in patients with complex and unusual anatomy.

**Learning points**

- Optimal result can be achieved with the transcatheter aortic valve implantation technique when treating complex anatomy such as bicuspid aortic stenosis and situs inversus totalis.
- Pre-operative planning and per-operative fluoroscopy image processing can provide valuable guidance when pursuing optimal technical result in complex and unusual anatomy.

## Introduction

Transcatheter aortic valve implantation (TAVI) can be challenging in case of complex anatomy. Transcatheter aortic valve implantation using the Edwards Sapien 3 (Edwards lifesciences, Irvine, CA, USA) valve has been recently reported to be an effective treatment of severe bicuspid aortic valve stenosis,^1^^,^^2^ and its positioning during the procedure is one of the main predictors of peri-prosthetic regurgitation.^3^ But the bicuspid valve’s angiographic appearance can be irregular and asymmetric and possibly lead to malpositioning. Furthermore, situs inversus yields unusual angiographic anatomical landmarks that could render the transcatheter heart valve’s (THVs) positioning even more hazardous. This report illustrates a successful procedure in a patient with both conditions after careful pre-operative planning and per-operative guidance by means of a novel software.

## Timeline

**Table:** 

Case	A 71-year-old man Symptomatic severe aortic stenosis of a bicuspid valve Situs inversus totalis
Day 1: Pre-operative investigations	Pre-operative multi-slice computed tomography: possibility of iliofemoral approach pre-operative planning through three-dimensional reconstruction
Day 2: Intervention	Transcatheter aortic valve implantation using a 26 mm Edwards Sapien 3 prosthesis while using per-operative fluoroscopy image processing for guidance Angiographic verification of the absence of periprosthetic leakage
Day 3–7: Post-operative care	Transthoracic echocardiography: no periprosthetic leakage Electrocardiogram: no atrioventricular block
Day 8: Patient discharge	Discharge at home
Day 90: Follow-up consultation	Improvement of symptoms

## Case presentation

**Figure 1:**
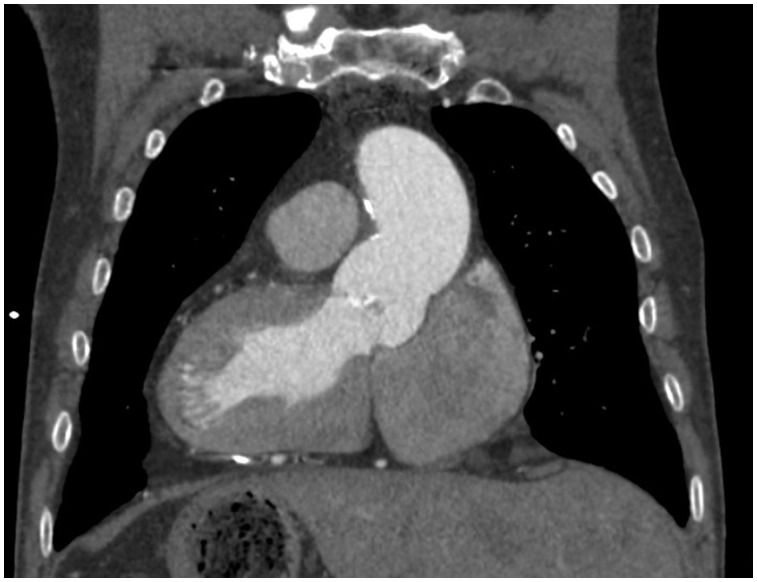
Transversal view of a computed tomography scan of the complete situs inversus.

**Figure 2:**
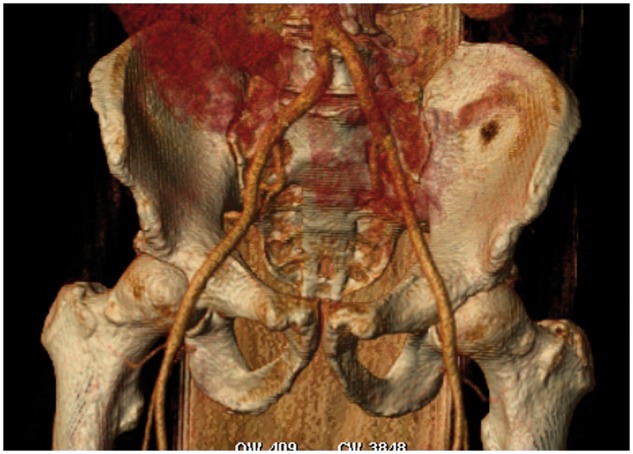
Three-dimensional computed tomography scan reconstruction of distal aorta, and it’s iliac and femoral branches. The usual transfemoral access was used in our patient.

A 71-year-old man was admitted to our hospital with dyspnoea due to severe aortic stenosis complicating a calcified type 0 bicuspid aortic valve. His medical history included diabetes mellitus, atrial fibrillation, chronic respiratory failure, and coronary artery bypass grafting surgery for left main artery stenosis, which uncovered a situs inversus totalis (*Figure 1*). On physical examination the patient had a systolic murmur predominantly located in the second left intercostal space on cardiac auscultation and bilateral crackles regarding the inferior parts of the lungs on pulmonary auscultation. Because of his medical history and a high Society of Thoracic Surgeons (STS) score of 11.9%, the Heart Team recommended TAVI rather than open surgery. The pre-operative multi-slice computed tomography (MSCT) evaluation of the aorta, and its branches confirmed patency of femoral arteries that allowed transfemoral approach (*Figure 2*) and absence of significant thoracic aortic aneurysm.

**Figure 3:**
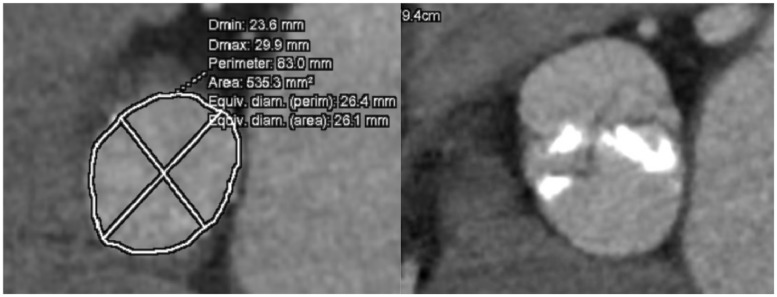
Computed tomography scan reconstruction of the aortic valve annulus of the bicuspid aortic valve.

**Figure 4:**
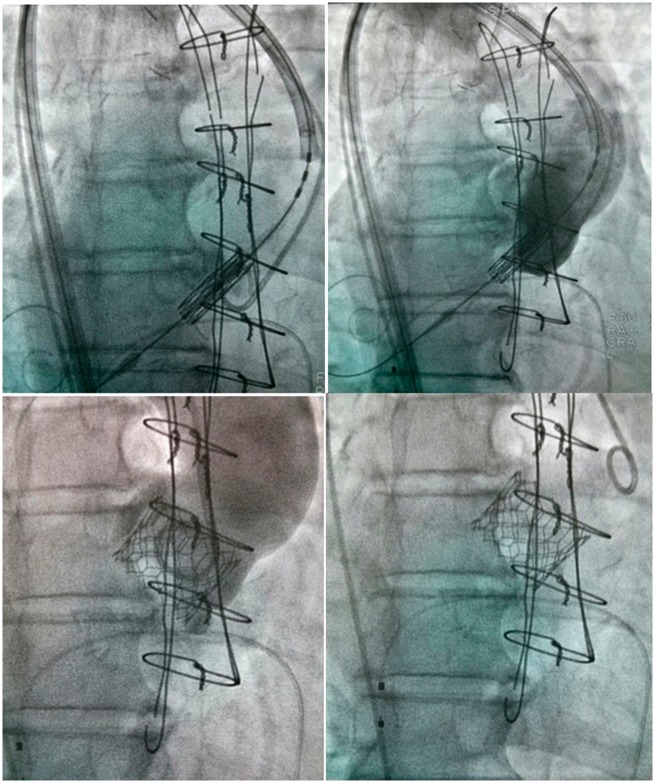
Per-procedural images with usual fluoroscopy.

**Figure 5:**
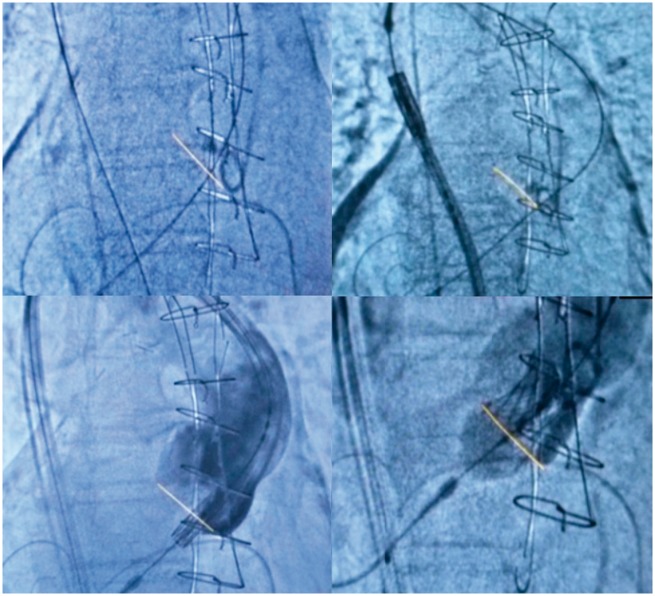
Per-procedural images with calcification enhancement and aortic annulus fusion on the fluoroscopy screen allowed by Valve Assist 2 software (Discovery IGS 730, GE Healthcare, Chalfont St Giles, UK).

Pre-operative sizing was performed with the new ValveAssist 2 (Discovery IGS 730, GE Healthcare, Chalfont St Giles, UK) image processing software. Comparatively to the standard fluoroscopy, the new software allows the projection of the MSCT-extracted, manually drawn virtual aortic annulus on the live fluoroscopy screen, and the enhancement of the aortic valve calcifications and aorta calcifications that are used as anatomical landmarks for operator guiding for the positioning of the THV during the procedure (*Figures 3–5*). A Sapien 3 (Edwards lifesciences, Irvine, CA, USA) 26 mm was directly implanted in a high position (regarding the leaflet extremities rather than the annulus, to reduce the risk of paravalvular regurgitation and need for permanent pacemaker), without post-dilatation, prosthesis constriction and no angiographic leak. The patient did not require permanent pacemaker implantation. A transthoracic echocardiography one week later confirmed an excellent result with no intra or paravalvular regurgitation, and the patient reported improved symptoms. In follow-up consultation 3 months after the procedure the patient reported improved symptoms and had no periprosthetic leak on transthoracic echocardiography.

## Discussion

To the best of our knowledge, this is the first report of TAVI in a patient with complete situs inversus and bicuspid aortic valve.^4^ Careful pre-procedural planning and precise THV implantation allowed the achievement of an optimal result despite complex anatomy in the case of our patient. Per-procedural fluoroscopy image processing with calcification enhancement and virtual aortic annulus fusion with new software such as Valve Assist 2 (Discovery IGS 730, GE Healthcare, Chalfont St Giles, UK) could help to achieve optimal results in selected patients with complex anatomy. Indeed, during TAVI the angiographic appearance of the cusps is irregular and asymmetric,^1^ which can lead to malpositioning, which in turn might increase the risk of paravalvular regurgitation^5^ and need for pacemaker implantation after TAVI.^6^ This is most evident in type 0 bicuspid valves where only two cusps exist and the classic angiographic orthogonal view of the three cusps of a tricuspid valve is absent.^7^ Furthermore, usual anatomical landmarks can be even more disturbed by the situs inversus totalis.^4^

We believe that pre-operative three-dimensional reconstruction and per-operative fluoroscopy image processing can provide precious guidance for TAVI in patients with complex and unusual anatomy. This case, along with previously published data,^1^^,^^4^^,^^7^ pleads in favour of feasibility of TAVI in patients with bicuspid aortic stenosis and situs inversus totalis with the Sapien 3 THV.

**Consent:** The author/s confirm that written consent for submission and publication of this case report including image(s) and associated text has been obtained from the patient in line with COPE guidance.

**Conflict of interest:** T.M. is proctor and consultant for Medtronic and Microport. A.S. has served as a consultant for Edwards and Medtronic. All other author declared no conflict of interest.
